# Reduced Mortality and Suicide Rates Among Israeli Veterans with PTSD Recognition: A Retrospective Analysis

**DOI:** 10.1192/j.eurpsy.2026.12003

**Published:** 2026-07-31

**Authors:** M. Frist, G. Lubin, N. Goldberger, Z. Haklai, A. Zeichner, T. Galil

**Affiliations:** 1 The Jerusalem Mental Health Center; 2 Statistical Center, The Israeli Ministry of Health; 3 Rehabilitation Department, The Israeli Ministry of Defense, Jerusalem, Israel

## Abstract

**Introduction:**

Research has shown a tendency towards higher mortality and suicide rates among veterans with PTSD compared to the general population.

**Objectives:**

To examine mortality, causes of death, and suicide rates among Israeli Defense Forces (IDF) veterans diagnosed with PTSD and formally recognized by the Ministry of Defense, and to compare these rates with those observed in the general Israeli population.

**Methods:**

This research compared total mortality rates for male PTSD-diagnosed veterans with national data for 1999–2023, for causes-of-death and suicides for 1999–2022 and for suicide attempts for 2004-2023. Age-standardized rates were calculated for overall mortality, causes of death, suicides and suicide attempts and rate-ratios and SMR’s computed compared to the general population.

**Results:**

Contrary to expectations and previous international findings suggesting higher mortality among veterans diagnosed with PTSD, our analysis revealed lower mortality rates for Israeli veterans recognized by the Rehabilitation Division in the Ministry of Defense as having a PTSD diagnosis compared with the general population. The rate ratios for male veterans diagnosed with PTSD only and those with PTSD and additional disabilities were 0.94 and 0.73, respectively. Mortality rates were lower for all causes apart from respiratory diseases and COVID-19. In particular, suicide mortality rates were non-significantly lower, SMR= 0.87 (95% CI = 0.46-1.48). However, suicide attempt rates were higher than the general population, the age-standardized rate ratio was 6.3 for veterans with PTSD only and 3.2 for those with PTSD and additional disabilities.

**Image 1:**
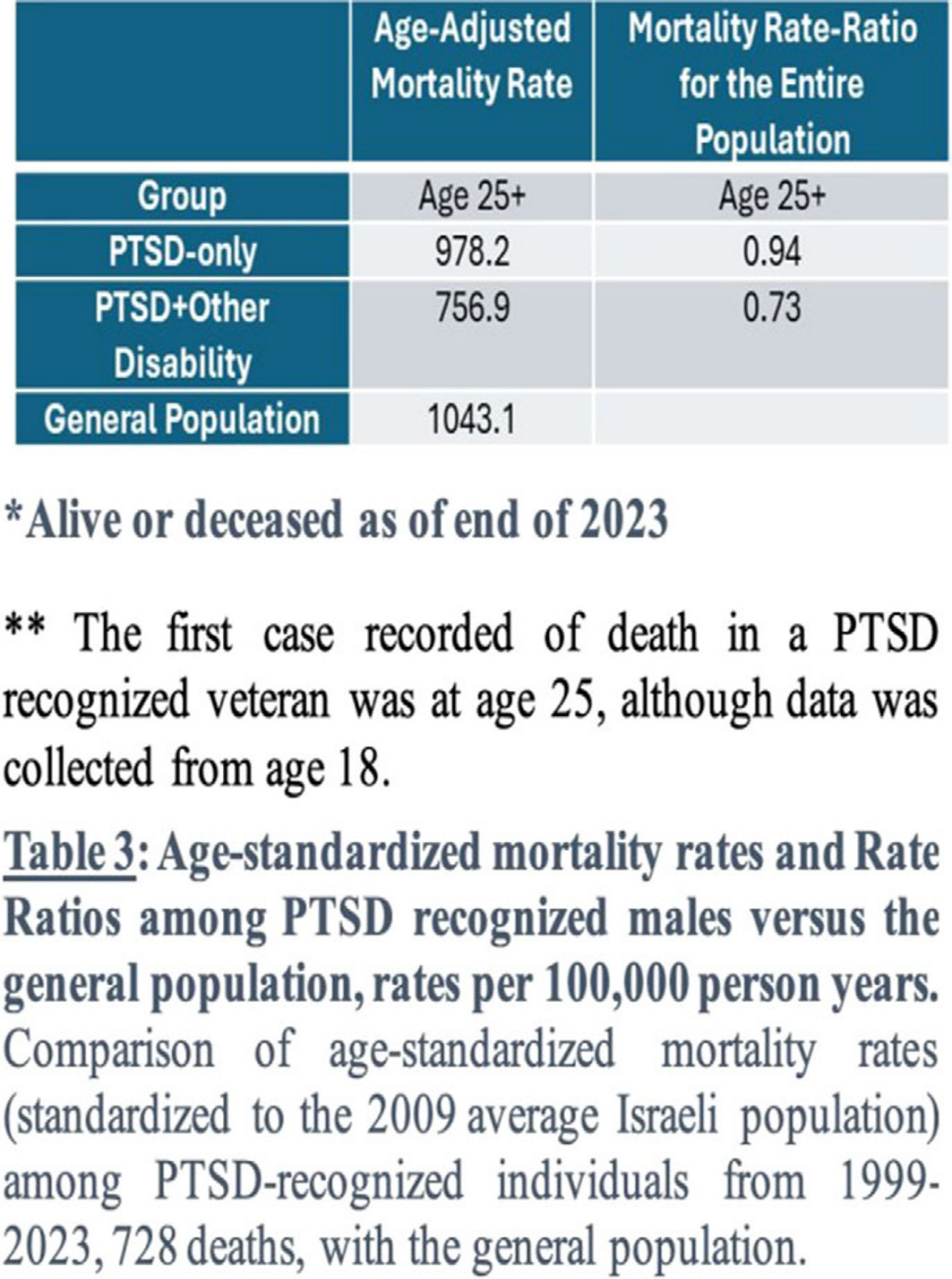
Image 1: Long description.

**Image 2:**
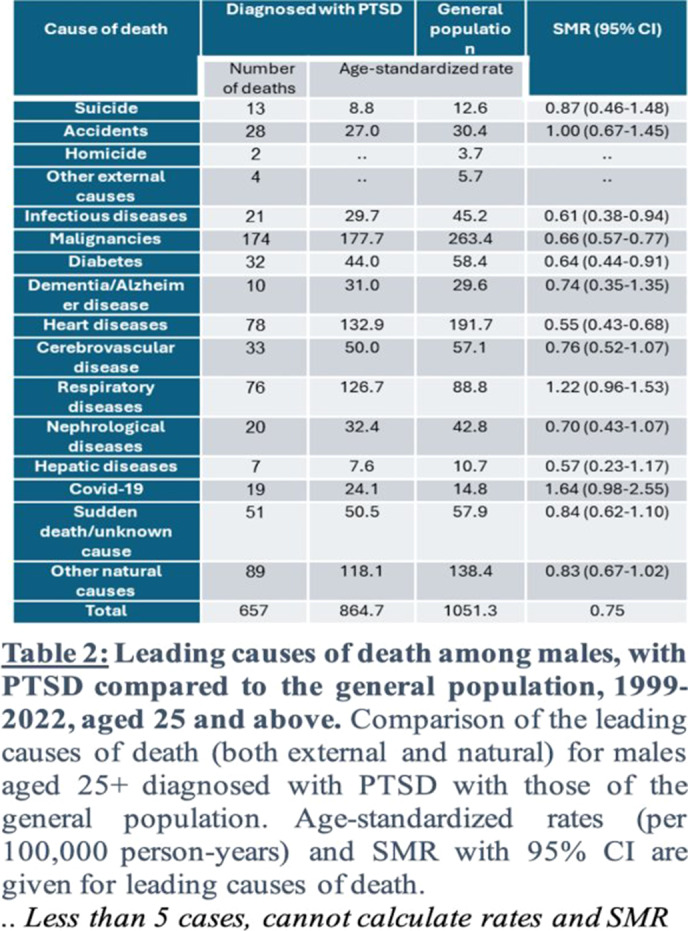
Image 2: Long description.

**Image 3:**
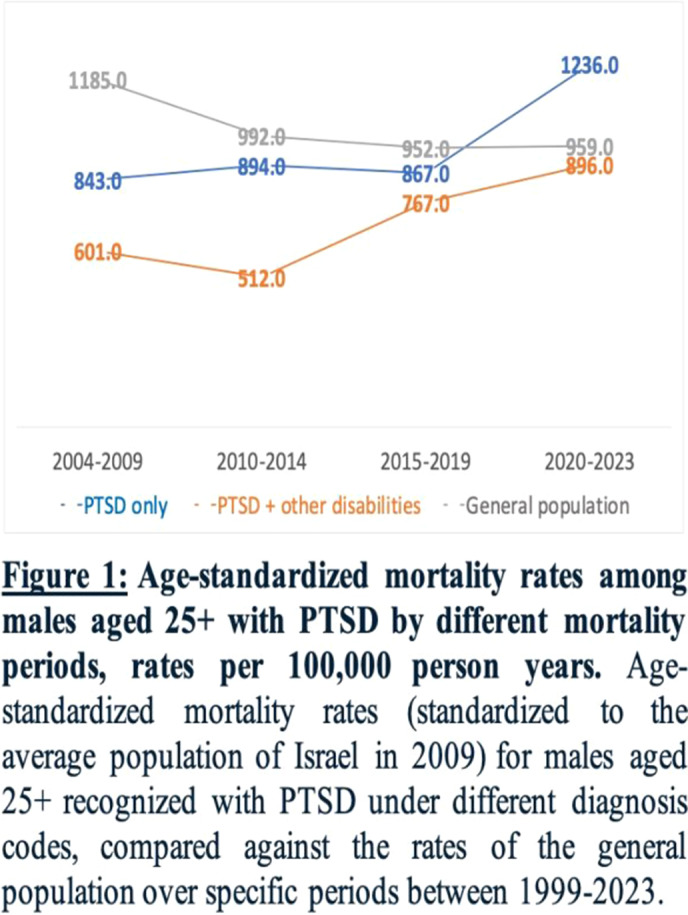
Image 3: Long description.

**Conclusions:**

Our findings show the importance of timely recognition of PTSD in Israeli veterans and continued targeted interventions, long-term psychiatric follow-up, and community-based mental health interventions for this population to maintain lower mortality and suicide rates and address their higher suicide attempt rates. They highlight unique Israeli social and organizational factors that may contribute to the lower rates found.

**Disclosure of Interest:**

None Declared

## Long descriptions

### Image 1: Long description

The table consists of three columns and four data rows.

Column headers from left to right are: Group, Age-Adjusted Mortality Rate (Age 25 plus), and Mortality Rate-Ratio for the Entire Population (Age 25 plus).

Row-by-row data:

- P T S D-only: Mortality rate of 978.2, Rate-Ratio of 0.94.

- P T S D plus Other Disability: Mortality rate of 756.9, Rate-Ratio of 0.73.

- General Population: Mortality rate of 1043.1, Rate-Ratio cell is empty.

Footnotes below the table:

- Single asterisk: Alive or deceased as of end of 2023.

- Double asterisk: The first case recorded of death in a P T S D recognized veteran was at age 25, although data was collected from age 18.

Table Title: Table 3: Age-standardized mortality rates and Rate Ratios among P T S D recognized males versus the general population, rates per 100,000 person years. Comparison of age-standardized mortality rates (standardized to the 2009 average Israeli population) among P T S D-recognized individuals from 1999-2023, 728 deaths, with the general population.

Navigate back to Image 1:.

### Image 2: Long description

The table contains four main columns: Cause of death, Diagnosed with P T S D (subdivided into Number of deaths and Age-standardized rate), General population (Age-standardized rate), and S M R (95% C I).

Data rows include:

* Suicide: 13 deaths, 8.8 rate vs 12.6 general, S M R 0.87.

* Accidents: 28 deaths, 27.0 rate vs 30.4 general, S M R 1.00.

* Homicide: 2 deaths, rate and S M R suppressed due to low cases.

* Other external causes: 4 deaths, rate and S M R suppressed.

* Infectious diseases: 21 deaths, 29.7 rate vs 45.2 general, S M R 0.61.

* Malignancies: 174 deaths, 177.7 rate vs 263.4 general, S M R 0.66.

* Diabetes: 32 deaths, 44.0 rate vs 58.4 general, S M R 0.64.

* Dementia/Alzheimer disease: 10 deaths, 31.0 rate vs 29.6 general, S M R 0.74.

* Heart diseases: 78 deaths, 132.9 rate vs 191.7 general, S M R 0.55.

* Cerebrovascular disease: 33 deaths, 50.0 rate vs 57.1 general, S M R 0.76.

* Respiratory diseases: 76 deaths, 126.7 rate vs 88.8 general, S M R 1.22.

* Nephrological diseases: 20 deaths, 32.4 rate vs 42.8 general, S M R 0.70.

* Hepatic diseases: 7 deaths, 7.6 rate vs 10.7 general, S M R 0.57.

* Covid-19: 19 deaths, 24.1 rate vs 14.8 general, S M R 1.64.

* Sudden death/unknown cause: 51 deaths, 50.5 rate vs 57.9 general, S M R 0.84.

* Other natural causes: 89 deaths, 118.1 rate vs 138.4 general, S M R 0.83.

* Total: 657 deaths, 864.7 rate vs 1051.3 general, S M R 0.75.

A footnote indicates that two dots (..) signify less than 5 cases, where rates and S M R cannot be calculated.

Navigate back to Image 2:.

### Image 3: Long description

The x-axis represents four time periods: 2004-2009, 2010-2014, 2015-2019, and 2020-2023. The y-axis represents mortality rates per 100,000 person years. Three lines are plotted:

* P T S D only (blue line): Starts at 843.0, increases slightly to 894.0, dips to 867.0, and then shows a sharp linear increase to 1236.0 in the final period.

* P T S D plus other disabilities (orange line): Starts at 601.0, decreases to 512.0, then shows a steady linear increase through 767.0 to end at 896.0.

* General population (grey line): Starts at the highest point of 1185.0 and shows a consistent downward trend through 992.0 and 952.0, ending at 959.0.

By the final period of 2020-2023, the P T S D only group has the highest mortality rate, surpassing both the general population and the P T S D plus other disabilities group.

Navigate back to Image 3:.

